# The Use of Contribution Analysis in Evaluating Health Interventions: A Scoping Review

**DOI:** 10.1177/01632787241281745

**Published:** 2024-09-08

**Authors:** David Buetti, Michael Fitzgerald, Cassandra Barber, Patrick R. Labelle, Isabelle Bourgeois, Tim Aubry, Erin Cameron, Claire E. Kendall

**Affiliations:** 1 School of Public Health, Université de Montréal, Canada; 2 152971 Bruyère Research Institute, Canada; 3 5211 Maastricht University, Netherlands; 4 University of Ottawa, Canada; 5 26627 Northern Ontario School of Medicine University (NOSM University), Canada

**Keywords:** contribution analysis, complex health interventions, scoping review, methodological challenges, stakeholder engagement

## Abstract

Contribution Analysis (CA) is a promising theory-based evaluation approach for complex interventions, yet its application in health interventions remains largely unexplored. To bridge this gap, we conducted a scoping review to examine the extent of such applications and the methodologies, strengths, and limitations of this approach in health programming. Our comprehensive search strategy was developed and used in 15 databases to identify peer-reviewed articles from 1999 to 2023 that focused on using CA to evaluate health interventions. We then implemented rigorous double- and triple-screening processes for abstracts and full-text papers, respectively. Data were extracted and narratively summarized. Our review found seven relevant studies, which showed that CA has been employed in health promotion programs, health policies, and targeted health issues such as nutrition, cardiovascular disease, substance misuse, and suicide prevention. The studies identified strengths of using CA, including its flexible impact evaluation approach, capacity to inform decision-making, and potential to enhance understanding of health programs and policies. However, challenges such as how to determine suitable evidence levels and how to best manage resource intensity were also identified. The limited number of studies indicates that CA is still a novel approach, whereas the variation in the reporting of the studies suggests that this approach could benefit from more standardized methods and detailed stakeholder engagement strategies.

## Introduction

Health interventions are planned actions and strategies to assess, maintain or improve health outcomes or determinants among individuals or populations (Kühne et al., 2015), which can differ in level (e.g., individual, community, or system), focus (e.g., prevention, treatment, or promotion), and stakeholders involved (e.g., health professionals, policymakers, or community members) (Craig et al., 2015; Skivington et al., 2021). These interventions can be considered complex when they involve multiple interacting components, contexts, and stakeholders that produce nonlinear and dynamic effects (Clarke et al., 2019; Craig et al., 2015; Kühne et al., 2015).

Experimental designs, such as randomized controlled trials (RCTs), are considered the gold standard for evaluating the results and impacts of health interventions (Hariton & Locascio, 2018). Their strength lies in their ability to isolate the effects of an intervention by comparing them with a counterfactual and minimizing the influence of confounding variables. In other words, experimental designs focus on the impact of interventions by separating them from their context to determine their net effect (Hariton & Locascio, 2018). However, this decontextualization can be a limitation for complex interventions, which often involve dynamic interactions within a specific setting and can also result in changes to the context itself (Barone, 2023; Forss et al., 2011; Skivington et al., 2021). These interactions can include feedback loops, emerging outcomes, and multiple pathways, making them difficult to capture using traditional experimental methods (Forss et al., 2011).

For example, in evaluating a community-based healthy eating initiative (intervention) that features cooking workshops and education, RCTs might be able to capture knowledge gained by participants (outcome) but would tend to overlook any social support networks that may develop (emerging outcomes). Participants might initiate potluck groups (feedback loop) emphasizing healthy eating and planning (multiple pathways) that cultivate community and accountability (dynamic effect). Such social dynamics, critical for sustained behaviour change, are challenging for RCTs to measure when not explicitly considered in the initial research question or design, potentially leading to underestimating the intervention’s broader impacts (Barone, 2023; Forss et al., 2011). As a result, when the focus is on specific pre-defined outcomes, experimental designs may not fully capture the real-world effectiveness of complex interventions or adequately explain the mechanisms through which they achieve their outcomes (Barone, 2023; Treasury Board of Canada, 2021). This is where Contribution Analysis (CA) becomes valuable, as it can help to systematically uncover the pathways and mechanisms contributing to both anticipated and unanticipated outcomes.

Contribution Analysis (CA) is a theory-based approach to evaluation that complements more narrowly causal methods. Rather than conclusively establishing a cause-and-effect relationship, this approach shifts the emphasis from attribution to understanding the intervention’s *contribution* to achieving outcomes (Bourgeois & Whynot, 2023; Mayne, 2019a). By underscoring this contribution, CA aims to diminish uncertainty surrounding the intervention’s influence on desired outcomes within a particular context (Delahais & Toulemonde, 2012; Mayne, 2019a). This approach is particularly valuable for complex interventions where multiple factors and stakeholders might influence outcomes, thus making it difficult to isolate a single cause (Delahais & Toulemonde, 2012; Mayne, 2019a). CA prioritizes a robust Theory of Change (ToC), a conceptual framework that visually outlines the hypothesized pathways for achieving the desired change, considering underlying assumptions and real-world influences (Mayne, 2019a). This focus on the ToC sets the stage for CA’s distinct approach to data collection. Unlike structural equation modelling (SEM) and other quantitative methods that primarily analyze relationships between variables based on statistical data, CA utilizes a mixed methods approach (Delahais & Toulemonde, 2012; Mayne, 2019a). This approach integrates both quantitative and qualitative data, enhancing the robustness of the evaluation through triangulation. By leveraging mixed methods, CA can more effectively assess an intervention’s effectiveness and the mechanisms behind its outcomes regardless of whether the efficacy of the intervention has been previously established. This ongoing process, informed by mixed methods data, provides a deeper understanding of how and why the intervention works, guiding future refinements for maximum impact (Delahais & Toulemonde, 2012).

CA was first codified by Mayne in 2001 and consists of six steps that guide users in building, testing, and refining a ToC that explains the causal links from the intervention to the outcomes: (1) defining the attribution problem, (2) developing a ToC, (3) collecting evidence on the ToC, (4) analyzing the evidence and the contribution story, (5) seeking more evidence if needed, and (6) improving the contribution story (Mayne, 2001). It emphasizes the need for explicit, well-articulated program theories with strong causal logic to understand how interventions lead to desired outcomes (Bourgeois & Whynot, 2023). The process is iterative, with the ToC continually being updated based on new evidence and feedback until a satisfactory level of confidence is reached (Delahais, 2023; Mayne, 2019b). CA also involves stakeholder engagement throughout the evaluation process to ensure their participation and ownership of the evaluation (Mayne, 2019a). The result is a rigorous, evidence-based ToC that accurately conveys the “contribution story” – a narrative account of the intervention’s impact (Mayne, 2019a). For complex interventions requiring multiple sub-ToCs, Mayne (2011) added a seventh step in which these sub-ToCs and their corresponding contribution stories are integrated into a comprehensive ToC.

Mayne (2011) also proposed three levels of CA - minimalist, direct, and indirect - depending on the intervention’s complexity and the assessment’s depth. All levels follow the same six-step process but differ in the type and amount of evidence needed. Minimalist CA is best suited for simple interventions with clear and measurable outcomes, where existing data and evidence are adequate for testing the contribution claim. The existing data and evidence may be rigorous and systematic but are not collected or explicitly analyzed for CA. Direct CA is for more complex interventions with multiple and diverse outcomes, where the contribution claim demands rigorous and systematic data collection and analysis customized to the CA questions and assumptions (Mayne, 2011). Indirect CA is for highly complex interventions with uncertain and emergent outcomes, where the contribution claim can only be deduced from the analysis of contextual factors and mechanisms (Mayne, 2011). Interventions in the health context often involve multiple levels, sectors and actors, for which CA would be a suitable evaluation approach. However, to our knowledge, reviews have yet to examine the literature on the application of CA to health interventions. Thus, it remains unclear how CA has been applied in this context.

### Objectives

We conducted a scoping review to examine the current state of knowledge on applying CA to evaluate health-related interventions. Our research questions were: (1) What types of health interventions have been evaluated using CA, and what was the purpose of using CA? (2) How has CA been implemented in the health context? (3) What are the strengths and challenges of using this approach? This review aims to reveal CA’s potential for diverse health interventions and inform future research efforts to refine and optimize its use by investigating these questions.

## Methods

### Study Design

We used Arksey & O’Malley’s (2005) scoping review framework and Peters et al.’s (2020) methodological guidance for scoping reviews to synthesize current knowledge on the use of CA in evaluating health-related interventions. To capture the earliest uses of CA, we included studies published in peer-reviewed journals from January 1999 to February 2023. We considered all studies that addressed the application or use of CA in health-related interventions with a clear and specific link to health behaviours or outcomes, including reflective and empirical studies, regardless of the study design. We adopted a broad conception of health-related intervention to ensure maximal representation. We excluded thesis or conference abstracts, prioritizing fully developed and peer-reviewed studies.

### Search Strategy

We searched 15 databases covering a range of disciplines on November 30, 2022, to identify pertinent studies: Academic Search Complete (EBSCOhost), APA PsycInfo (Ovid), Business Source Complete (EBSCOhost), CINAHL (EBSCOhost), Education Source (EBSCOhost), Embase (Ovid), ERIC (Ovid), Érudit, Global Health (EBSCOhost), International Bibliography of the Social Sciences (ProQuest), MEDLINE (Ovid), PAIS Index (ProQuest), ProQuest One Business (ProQuest), Sociological Abstracts (ProQuest) and Web of Science (Clarivate). An experienced research librarian initially tested two search strategies for use in MEDLINE (Ovid). One was a broad strategy using only “contribution analysis”; the other was a narrower strategy that combined evaluation-related keywords with the CA concept. Due to the similarities in the search results for both approaches, we chose the first strategy to capture all the relevant literature on CA, regardless of the keywords or terms used by the authors. The search strategy for MEDLINE (Ovid) was evaluated by another research librarian using PRESS guidelines (McGowan et al., 2016). In line with these guidelines, our secondary review prioritized the MEDLINE (Ovid) strategy, which served as our primary search strategy. Although the guidelines permit the review of additional databases, given that MEDLINE emphasizes health-related literature, we determined that a comprehensive and well-reviewed strategy in MEDLINE was adequate for the scope of this study. Strategies and keywords for all databases can be found in Additional File 1. Results were imported into Covidence (Veritas Health Innovation), an online tool that facilitates many screening steps in review projects, where duplicates were removed.

### Study Selection

Titles and abstracts were screened using Covidence based on three eligibility criteria: published in English or French, a journal article, and health-related intervention. As a pilot, the first and second authors independently screened twenty randomly selected references to ensure consistent application of eligibility criteria. Once the title and abstract screening process was completed, a pilot was set up to test the full-text screening eligibility criteria against seven randomly chosen articles. A fourth criterion was added to identify relevant content on CA, and seven references were tested to determine whether they should be included or excluded. The first, second, and third authors individually screened the full-text references for inclusion based on the eligibility criteria. Reviewers resolved any disagreements through discussion. The authors have no relationships or affiliations with core organizations promoting CA.

### Data Extraction

The first author developed a data-charting form in Excel to extract key variables from the included studies: study characteristics (e.g., authors, year, country, type of health program, and objectives); implementation of CA in the health context (e.g., approach used, adaptations made, methods and procedures used to build and refine the ToC, including data sources, collection methods, and analysis and interpretation techniques); and strengths and challenges in using CA. The research team reviewed the form for clarity, completeness, and relevance. Once validated, the first author extracted data from each study using the validated form, while the second cross-verified the extracted information for accuracy by comparing it with the sources.

### Data Analysis and Presentation

We performed a descriptive analysis of the frequency and distribution of different variables, such as types of studies, authors, year, and country. We also used content analysis methods suggested by Pollock et al. (2021) to examine the adaptations in applying CA, the procedures and methods for constructing and refining the ToC, and the strengths and challenges associated with using CA. This review’s findings are reported as per the PRISMA-ScR guidelines (Tricco et al., 2018) to ensure clarity and transparency.

## Results

**Figure 1.**
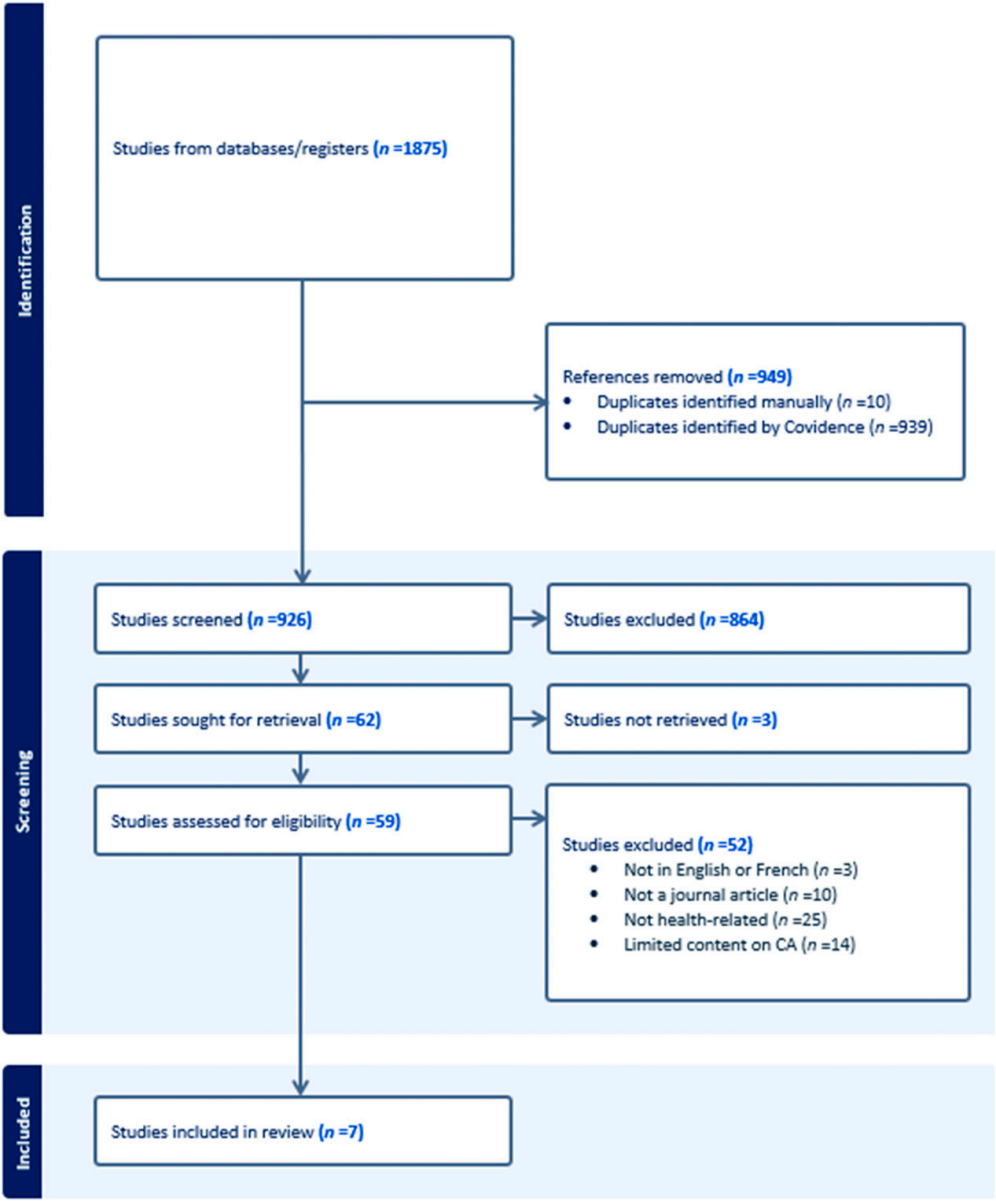
PRISMA Flow Diagram

Our search yielded 1875 citations, with 926 remaining after removing duplicates. After title and abstract screening, 62 studies were identified for full-text review. However, the full text for three of these could not be found. After reviewing the remaining 59 studies, another 52 were excluded, leaving seven studies for synthesis. The PRISMA flow diagram of the search and selection process is shown in Figure 1.

### Characteristics of Studies

Table 1 shows the characteristics of the seven studies included in the review. They were published between 2012 and 2020, mainly in English, and were authored by researchers from Canada, the United Kingdom, Australia, and the United States. Four of the studies were empirical, and three were reflective. Three of the four empirical studies reported on the methods used in conducting CA for health programs (Biggs et al., 2014; Downes et al., 2019; Riley et al., 2018), while one study assessed the feasibility and effectiveness of using CA to measure the impact of health impact assessment initiatives (Nour et al., 2017). The reflective studies examined the authors’ and evaluators’ experiences and challenges in applying CA to various health interventions.

**Table 1.** Characteristics of Studies

Authors	Year	Country	Type	Type of health program	Objective
Biggs et al.	2014	Australia	Empirical	Nutrition primary prevention program	Provide a practical example of CA and the Relevant Explanation Finder framework in a public health intervention.
Connolly	2016	UK	Reflective	Population-based cardiovascular health prevention intervention	Consider the congruence between CA and public value and examine its use in demonstrating value within partnership contexts during governance challenges.
Downes et al.	2019	USA	Empirical	Occupational safety and health policies	Explain the methods used to conduct CA in two research programs and their implications for practice and research.
Nour et al.	2017	Canada	Empirical	Health in all policies	Assess the feasibility and effectiveness of CA for assessing the effects of HIA approaches on the decision-making process of municipal stakeholders.
Livingston et al.	2020	UK	Reflective	Alcohol and drug policies	Reflect on the application of CA in evaluating alcohol and drug policies, focusing on its practical implementation and key learning points.
Riley et al.	2018	Canada	Empirical	Health research institute	Evaluate the impacts of a public health research center on policy changes through CA.
Wimbush et al.	2012	UK & Canada	Reflective	Varied	Argue the value of CA in theory-based evaluations by providing a structured approach to participatory evaluation planning, data analysis, and reporting.

### Types of Health Interventions

Two studies used CA to evaluate the effectiveness, implementation processes, and impacts of health promotion and prevention programs: a nutrition program that aimed to increase fruit, vegetable, and water consumption among primary school children (Biggs et al., 2014); and a national prevention program targeting cardiovascular disease mortality, morbidity, and risk factors (Connolly, 2016). Four studies used CA to evaluate the impact of health policies and research on health-related decision-making. These policies and research involved a national drug and alcohol substance misuse strategy (Livingston et al., 2019), the use of health impact assessment approaches on municipal decision-making processes (Nour et al., 2017), and the influence of health research on (a) the development and the adoption of three areas of tobacco control policy (Riley et al., 2018) and (b) morbidity and mortality (Downes et al., 2019). One study discussed four interventions, of which three were related to health: (a) a health charity to reduce the incidence of cancer and enhance the quality of life; (b) a national multi-component alcohol strategy to reduce alcohol-related harms; and (c) a local suicide prevention partnership to decrease the number of attempted and completed suicides (Wimbush et al., 2012).

### Implementation of CA in the Health Context

This section reviews how CA has been implemented practically, including adaptations made for the health context. Since CA relies on a robust ToC, it then outlines the methods and procedures used by studies to build the ToC before describing those used for testing and refining the ToC. Six studies used Mayne’s six-step approach (Biggs et al., 2014; Downes et al., 2019; Livingston et al., 2019; Nour et al., 2017; Riley et al., 2018; Wimbush et al., 2012), while one study used a four-step CA approach, comprising problem definition, model construction, monitoring and evaluation planning and data collection, and outcomes-focused performance/plausibility reporting (Connolly, 2016). Only one study specified the type of CA it applied, namely middle-level ‘Direct Influence CA,’ in evaluating the intermediate outcomes of health research programs on improved safety and health in workplaces (Downes et al., 2019).

#### Adaptations to CA for the Health Context

While CA is inherently iterative, two studies developed their ToC (step (2) and gathered evidence (step (3) iteratively, deviating from the sequential approach suggested by Mayne (Downes et al., 2019; Mayne, 2019a; Nour et al., 2017). This contrasts with the traditional linear approach, where the ToC is fully developed before evidence gathering begins. This deviation from the traditional linear approach might be due to the unique challenges of evaluating research programs or health interventions, as opposed to social programs for which the CA framework was originally designed (Nour et al., 2017). The authors reported that this approach enhanced the process’s efficiency by reducing the need for repeated data requests and creating a feedback loop between evidence collection and outcome verification. However, the specific reasons why this iterative approach was chosen over a sequential one are not fully articulated in these studies.

In another study, the authors found that integrating a Theory of Implementation/Action with a ToC significantly strengthened the CA (Wimbush et al., 2012). This integration was achieved by developing a results chain that outlined the implementation outcomes of the program. The final outcome of this chain was then connected to the ToC, which was centred on the expected impacts on the beneficiaries. This combined approach helped the authors address program implementation and delivery, lacking in the traditional CA method (Wimbush et al., 2012). The authors suggest that this improvement is especially beneficial for health interventions involving multiple partners, as it provides a comprehensive understanding of both the implementation process and the resulting impact of the program (Wimbush et al., 2012).

Riley et al. (2018) suggested a ‘good enough’ approach to conventional CA, aiming for a balance of relevance, rigour, and feasibility. Their study involved a team-based approach with a mix of internal and external members, nested theories of change that incorporated social science theories, and mixed-methods data. The authors concluded that there were three essential criteria for data collected for developing and testing the ToC: study relevance, reliable analysis quality, and diversity.

#### Methods and Procedures Used for Building the ToC

Six studies reported that they developed their ToC retrospectively (Biggs et al., 2014; Connolly, 2016; Livingston et al., 2019; Nour et al., 2017; Riley et al., 2018; Wimbush et al., 2012), with only Downes et al. (2019) also incorporating some activities near program lifecycle completion, demonstrating a prospective approach. Six studies provided visual representations of their ToCs, which integrated common elements such as inputs, activities, outputs, participation/reach, and outcome chains (Biggs et al., 2014; Downes et al., 2019; Livingston et al., 2019; Nour et al., 2017; Riley et al., 2018; Wimbush et al., 2012). Some studies also included rationale (Livingston et al., 2019), contextual and external factors (Downes et al., 2019; Livingston et al., 2019; Riley et al., 2018; Wimbush et al., 2012), alternative explanations (Biggs et al., 2014), assumptions (Biggs et al., 2014; Livingston et al., 2019; Riley et al., 2018), and hypothesized mechanisms of change (Nour et al., 2017).

Two studies utilized Bennett’s (1979) outcome hierarchy. This seven-component ToC model traces the progress from inputs to impacts: (1) input, (2) activities, (3) participation, (4) reaction, (5) knowledge, awareness, skills and attitude, (6) practice change, and (7) results (Riley et al., 2018; Wimbush et al., 2012). Both found this model beneficial for developing and streamlining result logic with stakeholders. One of these studies also categorized assumptions into behaviour change, capacity change and reaction assumptions, and added a temporal sequence of activities and outcomes (Riley et al., 2018).

Most studies provided details on the methods used to develop their ToCs, including participatory workshops (Connolly, 2016; Wimbush et al., 2012), document reviews (Livingston et al., 2019; Nour et al., 2017; Riley et al., 2018), discussions or consultations with stakeholders (Downes et al., 2019; Livingston et al., 2019; Nour et al., 2017; Riley et al., 2018), and working groups (Nour et al., 2017; Riley et al., 2018). One study also used scientific literature in ToC development by selecting a subset of relevant social science theories and creating a theory-based checklist that covered parameters, mechanisms, and contextual factors pertinent to their intervention (Riley et al., 2018).

Stakeholders commonly involved in the ToC development process included program staff (Connolly, 2016; Downes et al., 2019; Wimbush et al., 2012), management (Connolly, 2016; Downes et al., 2019; Wimbush et al., 2012), policy officials (Connolly, 2016; Nour et al., 2017; Wimbush et al., 2012), clinicians (Connolly, 2016), academics (Livingston et al., 2019; Riley et al., 2018), and evaluators (Downes et al., 2019; Wimbush et al., 2012). Two studies underscored the importance of involving a diverse group of stakeholders for a cross-level and cross-domain analysis and validation of the ToC (Connolly, 2016; Riley et al., 2018)

The level of detail about ToC development procedures differed across studies. For example, Biggs et al. (2014) developed a retrospective logic model for their intervention without providing any information on the process they used. In contrast, Riley et al. (2018) thoroughly described their consultation process, which involved iterative staff discussions, relevant document identification, ToC input gathering, and initial ToC feedback integration.

In participatory workshops, Connolly (2016) and Wimbush et al. (2012) utilized situational analysis to involve stakeholders in developing their ToC. The process encompassed collecting information about the context and current needs, the intervention and its execution, anticipated outcomes, and pertinent indicators for tracking progress and success. Winbush et al. (2012) found that group consensus and ownership were crucial for developing a ToC, ensuring shared, flexible outcomes that could accommodate multiple action theories, given the partners’ individual and joint activities. However, no details were given regarding the nature, duration, or frequency of these workshop sessions.

#### Methods and Procedures Used for Testing and Refining the ToC

##### Data Sources and Collection

All empirical studies collected both primary and secondary data to test their ToCs and assess the contribution claims of their interventions (Biggs et al., 2014; Downes et al., 2019; Nour et al., 2017; Riley et al., 2018). Interviews with internal and external stakeholders were a typical primary data source, providing insights into the observed outcomes’ causal mechanisms, contextual factors, and alternatives explanations (Biggs et al., 2014; Downes et al., 2019; Nour et al., 2017; Riley et al., 2018). One study used semi-structured interviews informed by the ToC and snowball sampling to capture the perspectives of critical actors involved in the policy process (Riley et al., 2018), while another used qualitative interviews to elicit the participants’ perspectives on program delivery, structure, implementation, and limitations, as well as their views on the ToC and its assumptions (Downes et al., 2019). Reflective papers also mentioned focus group interviews and participatory workshops as additional data sources (Connolly, 2016; Livingston et al., 2019; Wimbush et al., 2012).

Documentation reviews were another common data source for all empirical studies and aimed to complement the primary data sources with contextual information or serve as a distinct source for triangulation (Biggs et al., 2014; Downes et al., 2019; Nour et al., 2017; Riley et al., 2018). Documents reviewed included program records, administrative data, monitoring data, web statistics, reports, and research and evaluation reports (Biggs et al., 2014; Downes et al., 2019; Nour et al., 2017; Riley et al., 2018). Two studies also included scientific literature in their data collection scope to support the contribution claims and identify contextual factors (Livingston et al., 2019; Riley et al., 2018). Two studies focused data collection on a nested impact pathway, a specific outcome chain within the ToC (Riley et al., 2018; Wimbush et al., 2012). The choice of nested impact pathways stemmed from their feasibility and potential to generate new knowledge and valuable information that could inform future endeavours.

##### Data Analysis and Interpretation

All empirical studies applied data triangulation, corroborating different evidence sources to assess the consistency of findings and assumptions (Biggs et al., 2014; Downes et al., 2019; Nour et al., 2017; Riley et al., 2018). To address a perceived lack of data analysis methods in CA, Nour et al. (2017) employed argumentative discourse analysis. This approach explored linguistic markers within interviews that suggested change or causality.

All empirical studies employed multiple analysts or researchers to examine and interpret the data, thereby enhancing the objectivity of the findings (Biggs et al., 2014; Downes et al., 2019; Nour et al., 2017; Riley et al., 2018). Riley et al. (2018) explained their team approach to CA composition, which involved internal and external members with relevant expertise, increasing the rigour and objectivity of the analysis. Two studies also applied additional external validation methods, such as an independent panel to evaluate the contribution claim (Downes et al., 2019) and scientific theories to verify the contribution stories (Riley et al., 2018).

Some studies used or adapted tools to support data compilation and analysis during CA. Two studies incorporated Lemire et al.’s (2012) Relevant Explanation Finder Framework to systematically examine influencing factors and alternative explanations when analyzing the evidence and the contribution story (step 4) and seeking more evidence (step 5) (Biggs et al., 2014; Nour et al., 2017). This practical framework evaluates the evidence gathered regarding rival explanations of the intervention results using five criteria: certainty, robustness, prevalence, range, and evidence-based. Both studies found the Relevant Explanation Finder Framework useful for maximizing existing data and examining the supportive conditions that affect the relationship between the program mechanisms and the outcomes (Biggs et al., 2014; Nour et al., 2017).

Two studies, drawing upon Pawson et al.'s (2004) classification, organized the observed context into four categories: individual, interpersonal, institutional, and infrastructure. Riley et al. (2018) found the classification helpful in describing the intervention’s context and raising questions about the interaction of contextual factors. Their study applied Funnell and Rogers’ three approaches to evaluating the contribution stories: congruence, counterfactual comparison, and critical review (Funnell & Rogers, 2011). These techniques checked the alignment of the evidence and the ToC, compared the outcomes with and without the intervention, and examined the potential challenges and alternatives to the contribution claim. Riley et al. (2018) also used Morton’s ‘evidence of impact’ table, a structured tool for organizing and presenting evaluative evidence gathered on the intervention (see Morton, 2015). They found Morton’s table particularly beneficial in Step 4 of CA, aiding in identifying new data for collection in Step 5 and evaluating contribution stories in Step 6.

### Strengths and Challenges in Using CA for Health Interventions

#### Strengths

Studies identified four primary strengths of utilizing CA: (i) its flexible approach to impact evaluation, (ii) its ability to inform decision-making and refine interventions, (iii) its capacity to enhance conceptual understanding of health programs and policies, and (iv) its effectiveness in fostering ownership and appreciation of evaluation. Five studies highlighted CA’s flexibility in adapting the evaluation to the context and needs of each intervention (Biggs et al., 2014; Downes et al., 2019; Nour et al., 2017; Riley et al., 2018; Wimbush et al., 2012). They also noted its ability to adjust the depth and rigour of the analysis based on available resources and evidence (Biggs et al., 2014; Downes et al., 2019; Nour et al., 2017; Riley et al., 2018; Wimbush et al., 2012). CA also allowed the evaluation to focus on the most relevant and meaningful causal questions instead of measuring the impact of the intervention in isolation from other factors (Biggs et al., 2014; Downes et al., 2019; Nour et al., 2017; Riley et al., 2018; Wimbush et al., 2012). Downes et al. (2019) reported that the middle-level ‘Direct Influence CA’ enabled them to provide more specific and actionable recommendations targeting direct causal links between their intervention and its outcomes.

##### Informing Decision-Making and Improvements

Six studies indicated that CA offered valuable and actionable information to stakeholders, thereby influencing decision-making and enhancing the design and implementation of health programs and policies (Biggs et al., 2014; Connolly, 2016; Downes et al., 2019; Nour et al., 2017; Riley et al., 2018; Wimbush et al., 2012). For instance, Wimbush et al. (2012) demonstrated how CA findings informed future risk management strategies and improved program implementation, supporting strategic planning and performance management. Connolly (2016) reported that CA findings were used in developing a macro-level corporate strategy to inform decision-making and highlight the value of the organization’s programs and services.

##### Enhancing Conceptual Understanding of Health Programs and Policies

All studies demonstrated how CA informed the understanding of the impacts of health policies and interventions. This was achieved by differentiating these impacts from other explanations, identifying factors and mechanisms that affect health outcomes, and emphasizing learnings for future interventions (Biggs et al., 2014; Connolly, 2016; Downes et al., 2019; Livingston et al., 2019; Nour et al., 2017; Riley et al., 2018; Wimbush et al., 2012). For instance, Riley et al. (2018) reported that their CA helped identify enabling conditions that influenced observed policy changes, including the environment, institutions, interpersonal relations, and individuals. In another study, CA helped practitioners understand the social context of program delivery, enabling them to challenge the program’s conceptual logic and fidelity beyond output measurements (Biggs et al., 2014). A third study reported that the CA helped to explain why some areas of the strategy did not seem to result in the desired change as, from a contribution perspective, it was easier to consider the external factors that affected the intended outcomes (Livingston et al., 2019). This analysis included aspects that were outside the control of the delivery agents.

##### Increased Ownership and Appreciation of Evaluation

Four studies reported that stakeholder participation in CA led to increased ownership of results and a greater appreciation of and capacity for evaluative thinking (Connolly, 2016; Downes et al., 2019; Riley et al., 2018; Wimbush et al., 2012) and fostered a collaborative and trusting relationship between the evaluators and the stakeholders (Connolly, 2016; Wimbush et al., 2012). Wimbush et al. (2012) reported that using CA provided stakeholders with a common framework and vocabulary for analyzing organizational contribution in planning, reporting, and decision-making. However, most studies lacked details about the stakeholder engagement strategies used. Only Riley et al. (2018) provided comprehensive information on the composition of stakeholder groups, whereas Connolly (2016), Nour et al. (2017), and Wimbush et al. (2012) mentioned workshops and collaborative sessions as ways of engaging stakeholders in CA, without describing logistics and facilitation activities.

#### Challenges

##### Methodological Challenges

Five studies reported methodological challenges associated with using CA. Biggs et al. (2014) encountered difficulties determining the appropriate level of evidence needed to confidently connect the steps in their program logic to the desired outcomes. Livingston et al. (2019) also faced challenges in deciding which external factors to include in their analysis, as numerous factors could be considered relevant. Although some studies utilized tools like Lemire et al.’s (2012) Relevant Explanation Finder framework, they reported that their practical use could be more intuitive due to a lack of clear instructions, which adds complexity (Biggs et al., 2014; Nour et al., 2017). For example, Biggs et al. (2014) found it difficult to differentiate between influencing factors and alternative explanations using the Relevant Explanation Finder, as both involved similar information on mechanisms affecting observed outcomes. Specific challenges arise from the complex steps involved in the CA process, such as developing a ToC in multifaceted settings (Biggs et al., 2014; Livingston et al., 2019). Wimbush et al. (2012) highlighted a limitation in CA due to its ToC focus, neglecting vital program delivery aspects. They suggested refining CA’s methodology by including the theory of implementation/action for a more comprehensive assessment of program effectiveness (Wimbush et al., 2012).

##### Practical and Resource Challenges

All studies highlighted that CA is a time-consuming and resource-intensive process (Biggs et al., 2014; Connolly, 2016; Downes et al., 2019; Livingston et al., 2019; Nour et al., 2017; Riley et al., 2018; Wimbush et al., 2012). Downes et al. (2019) reported that their CA took seven months but would have been done more effectively if they had had 8–10 months. Engaging stakeholders, who often have competing priorities and face resource constraints, can further complicate matters according to some studies (Connolly, 2016; Downes et al., 2019; Livingston et al., 2019; Nour et al., 2017; Wimbush et al., 2012) For instance, Nour et al. (2017) reported that stakeholders who initiated the evaluation were replaced after elections and some elected officials did not agree to participate in scheduled interviews (Nour et al., 2017). Downes et al. (2019) could not access data within their evaluation timelines due to lengthy processes when securing data from federal agencies. Some studies also identified challenges in using CA retrospectively, leading to issues in data accessibility and quality (Downes et al., 2019; Livingston et al., 2019; Nour et al., 2017; Wimbush et al., 2012). These challenges were further compounded by the necessity for multiple rounds of data collection and potential stakeholder non-participation (Livingston et al., 2019; Nour et al., 2017).

## Discussion

This scoping review describes studies that used CA to evaluate health interventions. We found seven peer-reviewed studies using CA since 2012, which may indicate the novelty of CA as an evaluation approach in the health sector. Our findings indicate that CA has been utilized across a diverse range of health interventions, encompassing health promotion programs, health policies, and health research focused on decision-making. It has also been applied to strategies aimed at specific health issues such as nutrition, cardiovascular disease, substance misuse, and suicide prevention. This highlights the versatility and broad applicability of CA in the health sector.

Most studies referenced Mayne’s six-step CA method, which was anticipated given its foundational nature and widespread recognition in other sectors (Bourgeois & Whynot, 2023; Kotvojs & Shrimpton, 2007; Leeuw, 2023). However, none of the studies mentioned the seventh step that Mayne (2011) proposed for complex interventions. This may be because the contribution stories they developed did not use multiple ToCs. However, given the inherent complexity of most health interventions, the omission of the seventh step should perhaps be justified in future applications of CA. The studies identified several strengths and challenges associated with using CA to evaluate health interventions. The strengths included a structured evaluation framework for assessing the outcomes and impacts of health programs and policies, informing decision-making, and enhancing the understanding of health issues and contexts. In line with other collaborative evaluation approaches, CA also increased stakeholder engagement in evaluation by involving them in developing the ToC, identifying the contribution claims, and collecting and analyzing the evidence.

Beyond the time and resource demands of CA, studies identified the acquisition of high-quality and relevant data to support contribution claims as a key challenge. This was particularly evident in policy settings characterized by shifting priorities and high staff turnover. For instance, Nour et al. (2017) reported difficulties collecting data due to stakeholder turnover after elections. Similarly, Downes et al. (2019) faced lengthy federal agency data retrieval processes, impeding their evaluation timeline (Downes et al., 2019). Another challenge was the lack of standardized methods and procedures for applying CA, which can compromise the quality and credibility of the evaluation findings.

These challenges, while noteworthy, do not diminish the significance of CA. Instead, they highlight opportunities for further advancement and progress. Future research could explore the cost-effectiveness of CA compared to alternative evaluation approaches for health interventions. Such an analysis would equip potential users with valuable insights to determine when the potential benefits of using a deeper understanding of program effectiveness offered by a comprehensive approach such as CA might justify the associated investment. Some studies have also adopted streamlined CA processes with iterative cycles, minimizing data requests and fostering continuous feedback. Further exploration of these approaches holds promise for efficiency (Downes et al., 2019; Mayne, 2019a; Nour et al., 2017).

There was notable variation in how methods were presented in the empirical studies, possibly due to the current lack of reporting guidelines for CA. These variations make comparing how CA was applied in each study difficult. Standardized reporting guidelines or protocols for CA might improve rigour, transparency, and credibility and facilitate comparison, replication, and assessment of CA studies across different health contexts and interventions. We also found that most of the included studies needed more specific details on how they engaged stakeholders in different CA steps. Since stakeholder engagement is a crucial feature of CA, as it enhances relevance, validity and utilization of evaluation findings, documentation of stakeholder participation strategies from planning to dissemination of results would clarify how CA is being applied in this context and inform evidence-based practices for implementing CA with stakeholder involvement.

While our targeted search strategy across several databases was comprehensive, it was limited to academic literature. Given that CA is often used in program evaluations, future scoping reviews on CA in health should expand their scope to include grey literature. This could encompass unpublished evaluation reports and other non-peer-reviewed sources, which might offer valuable insights and details into how CA is used in practice settings.

## Conclusion

This scoping review examined the current state of knowledge regarding the application of CA in evaluating health-related interventions, along with the associated strengths and challenges. It provides a foundation for future research to enhance and improve the utilization of CA in health interventions. Despite its wide use in international development (Belcher & Palenberg, 2018) and public administration (Bourgeois & Whynot, 2023), few peer-reviewed studies on health-related evaluation using CA exist. Our review showed that CA is applicable to diverse health interventions, including health promotion programs, policies, and decision-making research, highlighting its broad adaptability.

Although CA can help understand how and why health interventions work as well as inform policy and practice decisions, several challenges were reported, such as the lack of standardized methods and procedures, the high demand for time and resources, and difficulty in accessing quality and relevant data in a timely manner. Future research efforts could explore the development of standardized reporting tools and streamlined CA processes to optimize its efficiency and facilitate wider adoption.
